# Identifying with all humanity predicts cooperative health behaviors and helpful responding during COVID-19

**DOI:** 10.1371/journal.pone.0248234

**Published:** 2021-03-10

**Authors:** Rodolfo C. Barragan, Nigini Oliveira, Koosha Khalvati, Rechele Brooks, Katharina Reinecke, Rajesh P. N. Rao, Andrew N. Meltzoff

**Affiliations:** 1 Department of Psychology, University of Washington, Seattle, WA, United States of America; 2 Institute for Learning & Brain Sciences, University of Washington, Seattle, WA, United States of America; 3 Paul G. Allen School of Computer Science and Engineering, University of Washington, Seattle, WA, United States of America; 4 Center for Neurotechnology, University of Washington, Seattle, WA, United States of America; University of Wolverhampton, UNITED KINGDOM

## Abstract

In the ongoing COVID-19 pandemic, public health experts have produced guidelines to limit the spread of the coronavirus, but individuals do not always comply with experts’ recommendations. Here, we tested whether a specific psychological belief—identification with all humanity—predicts cooperation with public health guidelines as well as helpful behavior during the COVID-19 pandemic. We hypothesized that peoples’ endorsement of this belief—their relative perception of a connection and moral commitment to other humans—would predict their tendencies to adopt World Health Organization (WHO) guidelines and to help others. To assess this, we conducted a global online study (*N* = 2537 participants) of four WHO-recommended health behaviors and four pandemic-related moral dilemmas that we constructed to be relevant to helping others at a potential cost to oneself. We used generalized linear mixed models (GLMM) that included 10 predictor variables (demographic, contextual, and psychological) for each of five outcome measures (a WHO cooperative health behavior score, plus responses to each of our four moral, helping dilemmas). Identification with all humanity was the most consistent and consequential predictor of individuals’ cooperative health behavior and helpful responding. Analyses showed that the identification with all humanity significantly predicted each of the five outcomes while controlling for the other variables (*P*range < 10^−22^ to < 0.009). The mean effect size of the identification with all humanity predictor on these outcomes was more than twice as large as the effect sizes of other predictors. Identification with all humanity is a psychological construct that, through targeted interventions, may help scientists and policymakers to better understand and promote cooperative health behavior and help-oriented concern for others during the current pandemic as well as in future humanitarian crises.

## Introduction

Early in the COVID-19 pandemic, the World Health Organization (WHO) provided health guidelines aimed at limiting the spread of the coronavirus. However, there have been varying degrees of individual compliance [1]. What psychological beliefs may influence whether or not an individual intends to follow the guidelines established by health organizations and governments? In daily life during the pandemic, individuals also face moral dilemmas about helping others. What factors might help predict people’s intention to come to the assistance of others during this humanitarian crisis, even at a cost to themselves? These questions raise important scientific as well as applied issues about cooperation, altruism, and concern for others.

Individuals exhibit widely different responses to social, ecological, and biological threats and crises [2]. When encountering these threats, some individuals become motivated to increasingly protect their personal interests while others seek to engage cooperatively and help others [3]. As in the 1918 flu pandemic, there has been widespread variation in responses by individuals in the COVID-19 pandemic, with some advocating for collective efforts to combat the virus and others advocating self-reliance [4–6]. Researchers have begun to examine the variation in the psychology of responses to the COVID-19 pandemic [7–10], because this variation has a key role to play in explaining consequential decision-making that affects both individuals and their societies [11–14]. Here, we argue that from a psychological perspective, a specific social-psychological representation of self and social relations—which has yet to be applied in the context of the novel coronavirus—is of vital importance in understanding individuals’ cooperative, helpful responses during the pandemic.

Sociological research has established that feelings or perceptions of connection toward strangers can promote and sustain helping behavior during a crisis [15], and recent social-psychological experimental work has systematized a means of quantifying peoples’ identification with others, including human beings one has never met [16, 17]. The tendency of some people to have deep feelings of connection and identification with “all humans” everywhere has sometimes been referred to as individuals’ perception that “all humanity is my ingroup” [16]. Indeed, it has been reported that this way of viewing the social world is correlated with a relative lack of ingroup favoritism, social dominance orientation, or right-wing authoritarianism, and instead is associated with a tendency to engage empathetically and altruistically with people beyond one’s neighborhood or nation, by extending commitments to the group of all humanity [16, 18]. Identification with all humanity is also positively related to impression management (e.g., the desire to appear in a positive light to others), but it is not redundant or reducible to this [16]. That is, in previous work it is specifically identification with all humanity, and not a number of other closely related personality and attitudinal predictors, that accounted for helpful behaviors toward strangers, as measured for example by the willingness to donate money to the victims of the 2010 Haiti earthquake or the 2011 Japanese tsunami [16, 19]. Moreover, the identification with other more constrained social groups—e.g., one’s own community or nation—although correlated with the all humanity construct did not predict the same outcomes [16]. For example, in the case of the tsunami, identification with all humanity predicted the amount of money pledged, whereas neither identification with community nor nation did so. This specificity has led to the proposal that individuals’ tendencies toward a generalized identification with the “family” of all humanity plays an especially strong role in calibrating one’s responses to strangers in need, including during major social-ecological crises [18].

Because beliefs facilitate action [20, 21], we hypothesized that peoples’ relative level of identification with all humanity may predict their responses to the COVID-19 pandemic. Notably, during COVID-19, people have the task of using their pre-existing belief system to decide whether to comply with experts’ and governments’ guidelines, as well as to decide on possible courses of action toward strangers who may be infected with the novel coronavirus. If a stranger in urgent need of assistance shows the signs of coronavirus infection, what psychological characteristics would predict helping that stranger even if it poses a risk to the self? We refer to this type of cooperative, helpful responding as “prosocial” responding in line with theorizing in social psychology [22].

We conducted a global study to investigate the following question: During the COVID-19 pandemic, does identification with all humanity [16, 18] predict cooperative health behavior (compliance with WHO guidelines) and also responses to everyday moral dilemmas in which people can show a helping tendency toward others with the disease? We expected that identification with all humanity would significantly predict a variety of pandemic-related cooperative and helpful behaviors that would be robust to controls for other relevant variables (age, gender, and other demographic, contextual, and psychological factors).

We used five outcome measures. Our first and most important concerned the respondents’ compliance with the WHO’s pandemic-related health guidelines that were in place and internationally circulated at the time of our study before vaccines were available (e.g., social distancing, see Methods and materials). We also constructed COVID-related moral dilemmas building on previous research on moral decision making [23, 24]. Our second outcome measure was thus formulated as a COVID-19 analog to the public good game (PGG) used in game theory [25]: Respondents were asked to suppose that a local hospital is in urgent need of face masks and that their family had 10 masks available for their own use—how many would they donate? Third, respondents were asked about encountering a person who shows the signs of COVID-19 on the side of a lonely road. Would our respondents leave this person alone or take them to the hospital, thus risking self-exposure to the virus? Fourth, would respondents go shopping for a family that needs food despite strong “stay-at-home” guidelines and a personal risk of exposure to COVID-19? Fifth, we asked respondents if they would call and wait for an ambulance for a person showing COVID-19 symptoms and having trouble breathing, despite there being other bystanders who could help? As such, our outcome measures tapped individual differences in respondents’ inclinations toward cooperative, helpful behavior during the COVID-19 pandemic.

## Methods and materials

### Participants and recruitment

We used the online experiment platform (https://www.labinthewild.org/studies/covid-dilemmas/ [26]) to launch a global online survey on COVID-related health behaviors and moral dilemmas. The research was conducted with the approval of the Human Subjects Institutional Review Board (IRB) at the University of Washington, approval number: 00006878. All methods were performed in accordance with the relevant guidelines and regulations. All respondents gave online, electronic consent for participation before completing the survey. The survey was available in English and five other languages. The language was automatically adjusted depending on the participants’ browser-determined language, defaulting to English. The demographics were assessed at the beginning of the survey and the other categories of variables were presented in four blocks (contextual factors, cooperative health behaviors, moral dilemmas, identifications), which were presented in a random order across participants.

We preset the endpoint of data collection to be when we received 100 participants each in at least 10 countries. After launching the survey on April 14th, 2020, this pre-established criterion was achieved on June 17^th^, 2020. In that window we received 2,566 responses, which came from 87 countries/territories and from all continents. After 29 exclusions based on missing data on focal variables, the final analytic sample was 2,537 respondents (25.42% U.S.A, 13.40% China, 6.98% South Africa, 6.54% Germany, 5.20% U.K., 5.05% Philippines, 4.53% India, 4.26% Brazil, 4.06% Spain, 3.90% Canada, 20.65% Other).

### Analytic strategy

We tested our hypothesis with generalized linear mixed models (GLMM) with respondents (Level 1) nested within their countries (Level 2), which controlled for country membership to allow for generalization across countries. The nesting within countries was accomplished by treating any country with at least 10 respondents as a unit (resulting in 20 country units), and grouping the remaining countries with 9 or fewer respondents as a single “other” unit. Thus, there was a total of 21 Level 2 units. (For completeness, we also checked dropping participants from the “other” category, leaving 20 Level 2 units, see S1 Fig in S1 File. The pattern remained essentially unchanged from the results obtained with the full analytic sample of *N* = 2537, which are reported in the Results section.)

The models included 10 predictor variables, including nine that can be considered control variables (demographics, etc.) and our hypothesized predictor of identification with all humanity. To aid the interpretation of the GLMM results, we converted continuous or scale variables to z-scores (*M* = 0, *SD* = 1) and used effect coding for dichotomous variables. After inspection of the data distributions, the cooperative health scores and the number of masks donated were fit with Poisson distributions (applicable for right-skewed data and count data, respectively). The other three outcome measures assessing prosocial responding (helping at roadside, shopping for a family, and calling an ambulance), which were each binary data, were fit with Bernoulli distributions. We calculated effect sizes for each predictor by dividing each regression coefficient (beta) by the product of *SE* beta and square root of 2537 (the sample size), which may be interpreted as Cohen’s *d*. The R lme4 package was used to model each of the outcomes with full maximum likelihood estimation (allowing comparison of model fit). We conducted likelihood ratio tests to evaluate the difference between two models for each outcome measure: (a) Model 1: A model with only the nine control variables and (b) Model 2: A model including the nine control variables plus the predictor we hypothesized to have the major effect, identification with all humanity. Using this analytic strategy, significance for the likelihood ratio tests would indicate that identification with all humanity significantly improved model fit.

### Outcome measures

The five outcome measures provide for an examination of whether people’s degree of identification with all humanity is linked to their cooperative, helpful responses to the global health crisis.

#### (1) Cooperative health behaviors

We asked respondents their likelihood of complying with the key health behaviors that were identified and internationally circulated by WHO in April 2020 (when the study started). The survey prompted respondents to report, if they were to leave their house the next day, how likely they would be to: (i) wash their hands thoroughly and frequently, (ii) cover their mouth when coughing or sneezing, (iii) social distance from others, and (iv) avoid touching their own face. Respondents’ answered each of the four on a scale of 1 (“Not at all likely”) to 5 (“Very likely”), which resulted in a score ranging from 4 to 20 for each respondent.

#### (2) Donating masks

Respondents were asked about donating a valuable pandemic resource. Respondents were given a scenario in which they had 10 single-day-use face masks for their family of three, and asked how many masks they would decide to donate to a local hospital in urgent need of face masks. Respondents answered with a number ranging from 0 to 10.

#### (3) COVID-19 afflicted person at roadside

Respondents were asked whether or not (binary response), while driving on a lonely road with limited cellphone signal, they would choose to leave a person whom they encountered by the side of the road, despite this individual exhibiting all the symptoms of COVID-19.

#### (4) Grocery shopping for a family

Respondents were asked whether or not (binary response) they would go shopping for a family that needed food despite strong “stay-at-home” guidelines and a personal risk of exposure to COVID-19.

#### (5) Call an ambulance with bystanders present

Respondents were asked whether or not (binary response), when walking on a street, they would call and wait for an ambulance for a person showing signs of COVID-19 and having trouble breathing.

We also asked respondents three questions that were not connected to pandemic cooperation or helping others (on medical decision making), and these are not included in this paper.

### Predictor variables

We examined 10 predictor variables. Probing the relations between these multiple predictors and the outcome measures allowed us to check for alternative explanations of our hypothesized relation between identification with all humanity and the five outcomes (cooperative health behaviors, donation of masks, helping responses in the case of encountering afflicted person at roadside, shopping for a family, calling an ambulance).

#### Time in days (1 item)

To investigate whether there was an effect of time (days since start of the study), we tabulated the day of individual’s responses in relation to the start of data collection. Data collection started on April 14, 2020 and lasted 64 days. We entered this variable in our analyses to capture the date that the participant took the test, relative to the April 14^th^ start date.

#### Contextual factors (3 items)

We also measured three different contexts, and each served as a predictor variable. First, we measured local societal *restrictions* related to COVID-19 on a set of five yes/no questions. These items were: (i) “My city or region is under ‘stay-at-home’ orders by the local or national government,” (ii) “To leave the house, one has to fill in an official document to justify it,” (iii) “All stores in my city except for ‘essential businesses’ are closed,” (iv) “Schools in my city are closed,” and (v) “The country I live in has closed some of its borders.” Respondents could also report “none of the above.” This resulted in a score ranging from 0 to 5 for the restrictions variable. Second, we measured the availability of *testing* for COVID-19 through a one-item dichotomous measure (yes/no, with yes assigned as +1): “Testing for COVID-19 is easily available.” Third, we measured respondents’ perceptions of their personal *risk* based on the virus by asking: “Do you consider yourself to be at high risk for severe illness if infected with COVID-19?” Respondents could choose no, not sure, or yes (+1 assigned for yes and -1 for no / not sure, for statistical analyses).

#### Demographic factors (3 items)

We measured three basic demographic variables: *Age*, *gender*, *and educational attainment*. Respondents were prompted to report their age. The respondents identified their gender from the options of female, male, non-binary, and other. Because of insufficient *n* (< 2% for non-binary and other), this was collapsed into a dichotomous measure of female (+1) and non-female (-1) for statistical analyses. Respondents could also chose their educational attainment from nine options: No formal education, incomplete primary school, complete primary school, incomplete secondary school or high school, complete secondary school or high school, some university education, university-level education with degree, incomplete graduate or professional school, or complete graduate or professional school. For analytic purposes, because the *n*s were too small to retain nine categories, we collapsed this to a dichotomous measure of having a university degree (+1) or not (-1) for statistical analyses.

#### Related psychological factors (2 items)

Identification with *community* and identification with the *nation* as closely related psychological constructs to our key variable of identification with all humanity [16]. Following previous research [27], we used a shortened four-item measure, each rated with a 1 (“Not at all”) to 5 (“Very much”) scale. The four items—each asked separately in relation to community or nation—were: (i) “How much do you want to be a responsible citizen of your community (identification with community)/your country (identification with nation)?”, (ii) “How much do you believe in being loyal to my community (identification with community)/my country (identification with nation)?”, (iii) “How much would you say you care (feel upset, want to help) when bad things happen to people in my community (identification with community)/my country (identification with nation)?”, and (iv) “When they are in need, how much do you want to help people in my community (identification with community)/people in my country (identification with nation)?” The community identification scale was internally consistent (Cronbach’s α = 0.81), as was the national identification scale (Cronbach’s α = 0.80). The score analyzed was the mean of the four items for each scale, thus scores ranging from 1 to 5.

#### Identification with all humanity (1 item)

Identification with *all humanity* was measured similarly to identification with community and nation. While originally measured using a nine-item scale [16], the four items we used were have been shown to be both reliable and predictive of behavior [27]. The four questions were each measured on a 1 (“Not at all”) to 5 (“Very much”) scale: (i) “How much do you want to be a responsible citizen of the world?”, (ii) “How much do you believe in being loyal to all humanity?”, (iii) “How much would you say you care (feel upset, want to help) when bad things happen to people all over the world?”, and (iv) “When they are in need, how much do you want to help people all over the world?” The scale was internally consistent (Cronbach’s α = 0.84). The score analyzed was the mean of the four items, thus a score ranging from 1 to 5.

## Results

Descriptive statistics are provided in Table 1. Zero-order correlations among the 10 predictor variables are shown in the S1 Table in S1 File. We tested our main hypothesis with generalized linear mixed models (GLMM) that included 10 predictor variables for each of the five outcome measures (a WHO cooperative health behavior score, plus responses to four moral, helping dilemmas). In these models, respondents were nested within their countries; in so doing, we controlled for country membership in all analyses so that results would be generalizable across countries. Given that many factors may predict the outcomes, we took into account other variables in addition to our key all humanity psychological predictor: (i) time (in days) since start of data collection (to adjust for temporal variations in the data), (ii) restrictions enacted by local government, (iii) availability of testing, (iv) perceived risk for severe COVID-19 illness, (v) age, (vi) gender, (vii) education, (viii) identification with community, (ix) identification with nation, (x) identification with all humanity. In sum, the GLMM analytic approach allowed us to assess whether our hypothesized all humanity variable significantly predicted the outcomes while controlling for the nine other variables (see above “Analytic strategy”).

**Table 1:** Descriptive statistics for predictor and outcome variables, including Cronbach’s alpha for scale scores (*N* = 2537).

Variables	*M* or %	*SD*	Range	α
**Predictors**				
Days	27.92	20.74	0–64	
Restrictions	2.66	1.52	0–5	0.70
Testing (% available)	25.78			
Risk (% high risk)	22.78			
Age (years)	39.52	16.01	18–86	
Gender (% female)	66.06			
Education (% university degree)	72.29			
Community	4.29	0.71	1–5	0.81
Nation	3.99	0.82	1–5	0.80
Humanity	4.04	0.83	1–5	0.84
**Outcomes**				
Cooperative health behaviors	17.76	2.62	4–20	0.70
Masks donated	4.95	3.29	0–10	
Helping person at roadside	72.84			
Shopping for a family	75.56			
Calling an ambulance	90.97			

**Fig 1:**
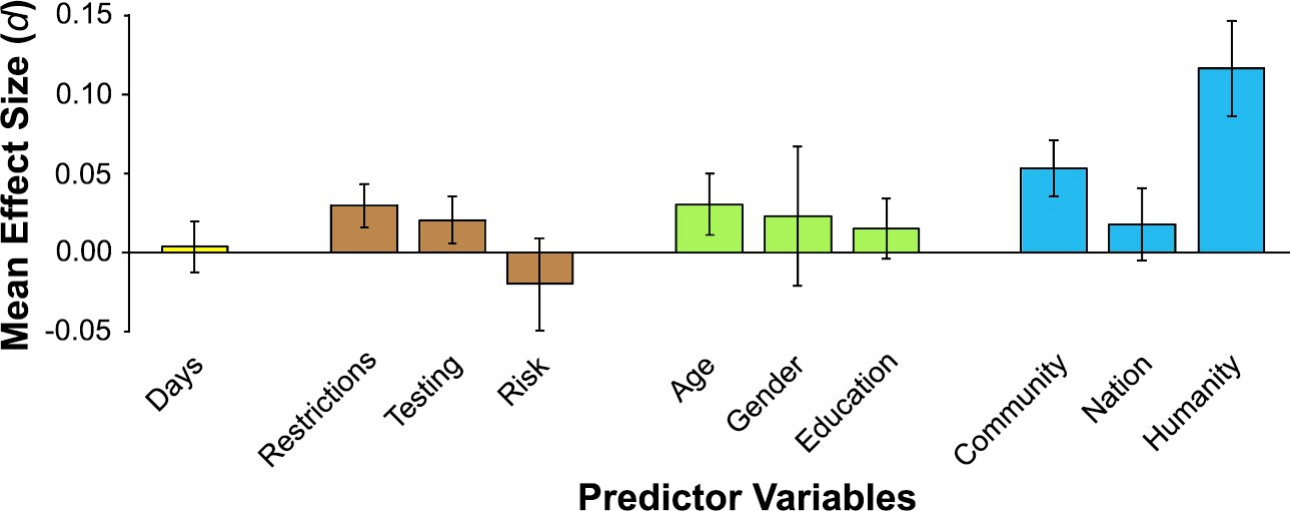
Mean effect sizes (estimated Cohen’s *d*) across five outcomes for each predictor variable. The predictors are grouped into five classes (indicated by color): time/days since survey start (yellow), contextual factors (brown), respondent demographic characteristics (green), two psychological identification variables and our hypothesized predictor of identification with all humanity (blue). Positive values indicate that the predictor is associated with higher outcome scores. Error bars represent the standard error of the mean effect size.

**Fig 2:**
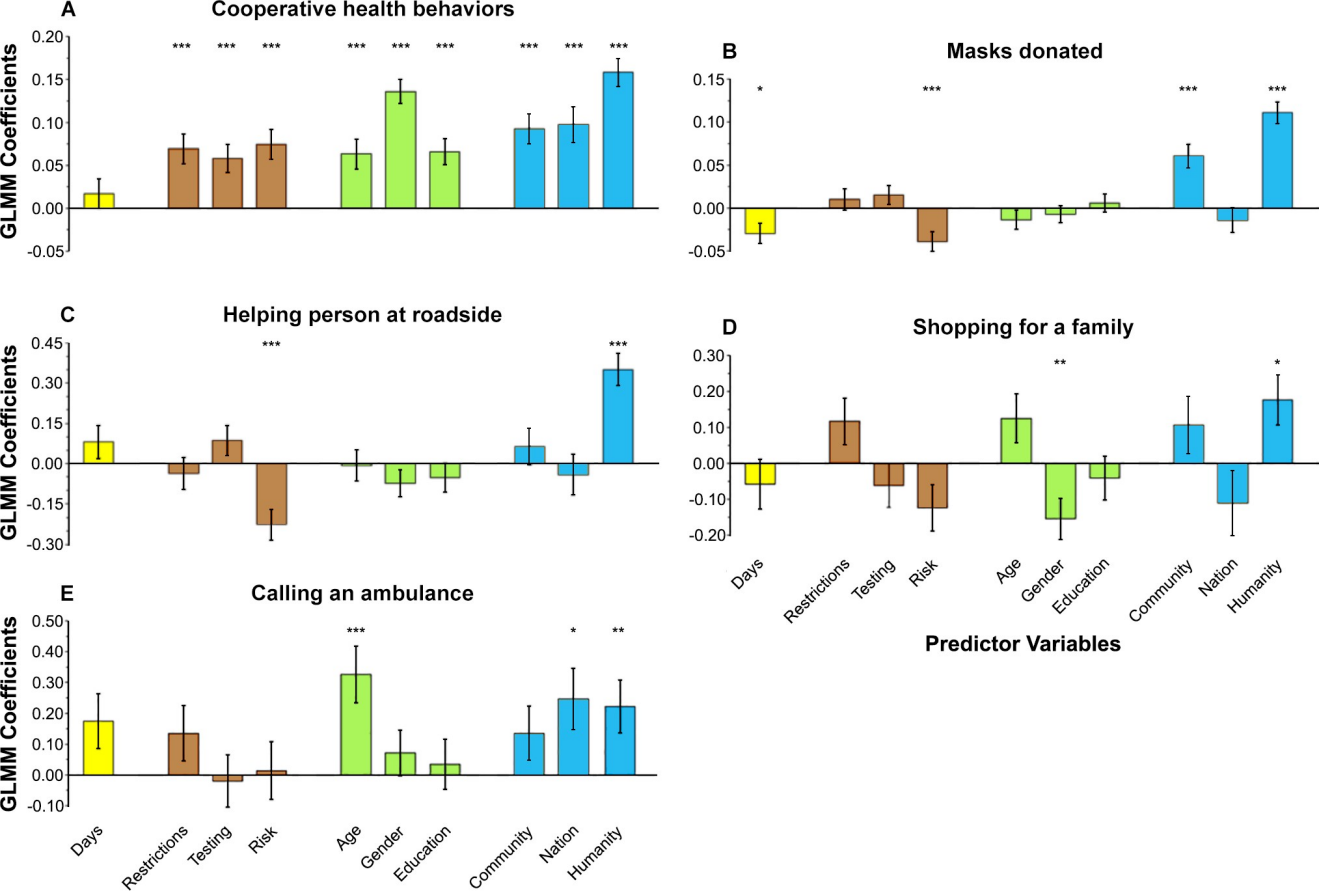
Results for each of five outcomes. **(A)** Cooperative health behaviors (log score), **(B)** Masks donated (log count), **(C)** Helping a person at roadside (log of odds), **(D)** Shopping for a family (log of odds), and **(E)** Calling an ambulance (log of odds). Plotted on the y-axis is the magnitude of GLMM regression coefficient estimates. The predictor variables listed in the x-axis are grouped into five classes (indicated by color): time/days from survey start (yellow), contextual factors (brown), respondent demographic characteristics (green), two psychological identification variables and our hypothesized predictor of identification with all humanity (blue). Positive coefficients indicate that the predictor is associated with higher outcome scores. Error bars represent the standard error of the regression coefficients. *P < 0.05. **P < 0.01. ***P < 0.001.

Analyses showed that the identification with all humanity variable significantly predicted each of the five outcomes while controlling for the nine other variables. The results showed that identification with all humanity significantly predicted cooperative health behaviors, *b* = 0.16, *SE* = 0.02, *z* = 9.76, *P* = 1.7 × 10^−22^. The all humanity variable significantly predicted the number of masks donated, *b* = 0.11, *SE* = 0.01, *z* = 8.78, *P* = 1.6 × 10^−18^. Identification with all humanity also significantly predicted the outcomes for helping a person on the side of the road, *b* = 0.35, *SE* = 0.06, *z* = 5.71, *P* = 1.1 × 10^−8^; shopping for a family, *b* = 0.18, *SE* = 0.07, *z* = 2.51, *P* = 0.012; and calling for an ambulance, *b* = 0.22, *SE* = 0.09, *z* = 2.60, *P* = 0.009. Thus, for each outcome, identification with all humanity was associated with higher outcome scores. Fig 1 graphically depicts the key findings: The mean effect size for identification with all humanity was more than twice as large as any of the other predictors. Fig 2 shows that identification with all humanity was the only variable that significantly predicted all five outcomes.

The importance of identification with all humanity as a predictor was also statistically assessed by comparing two different generalized linear mixed models: (i) one included all nine control variables and (ii) the other had the same nine but added the hypothesized predictor of identification with all humanity. As shown in Table 2, the improvement in model fit when adding all humanity to the model was significant for each of the five outcomes (i.e., for cooperative health behaviors, masks donated, helping at roadside, shopping for family, calling an ambulance).

**Table 2:** Likelihood ratio tests (LRT) showing that identification with all humanity (Model 2) significantly improves model fit over control variables (Model 1) for each of the five outcomes.

Outcome	LRT (*df* = 1)	*P*
Cooperative health behaviors	-93	< 0.0000000001
Masks donated	-78	0.0000000007
Helping person at roadside	-33	0.000000009
Shopping for a family	-7	0.008
Calling an ambulance	-6	0.014

## Discussion

The converging results from the effect sizes (Fig 1), the magnitude of the regression coefficients (Fig 2), and the comparison of Model 1 and 2 (Table 2) suggest that, during the COVID-19 pandemic, there is a consistent and powerful role for identification with all humanity in predicting self-reported cooperative and helpful responding. When identification with all humanity was included alongside other variables that may reasonably relate to pandemic responses, these other variables had a less consistent role in predicting prosocial responding, which extends and generalizes previous social psychology work using the identification with all humanity construct [16–19]. Based on these patterns, it may be useful to design studies to examine the degree to which identification with all humanity predicts not only self-reported intentions but people’s actual behavior during the current and future pandemics.

It is notable that, of the three psychological identification variables evaluated in this research (i.e. identification with community, with nation, and with all humanity), it was identification with all humanity that more strongly predicted positive outcomes than these other related variables. Yet, it is also evident that identification with one’s own community also played a role (Fig 1). This suggests that peoples’ drive to respond prosocially to close others accounts for some aspects of responses to the pandemic, which warrants further investigation. Nonetheless, the bulk of our respondents’ prosocial responding in the context of our COVID-19 study was linked to identification with humanity not just those from one’s close-in community.

It is also of interest that identification with one’s own nation played less role in predicting the outcomes than identification with community or all humanity (see Figs 1 and 2). Traditionally, it has been reported that strong identification with one’s nation—“nationalism”—is tied to parochial ingroup favoritism and hostility toward the outgroup, e.g., opposition to immigration [28]. That is, there are obstacles to the psychological disposition of favoring the well-being of different human groups. In the context of COVID-19, research has begun on how national identity may influence behavior in the pandemic [29]. The current data add to this by showing that when different identifications (community, nation, all humanity) are assessed in the *same* individual, cooperative health behavior and helpful responding are more linked to a generalized feeling of connection to the larger “family” of humanity—to people everywhere—than to an identification with one’s own nation in particular. The tendency toward promoting others’ well-being during the pandemic may have more to do with individuals’ tendencies to feel connected to the human species as a whole, rather than to one’s more narrow geopolitical identity—which is consistent with the original work on identification with all humanity [16, 18].

The current findings point to future experimental work of potential scientific and societal importance as well as some limitations. First, we acknowledge that the current study was correlational, and this precludes causal inferences. Notwithstanding, we consider it intriguing that among all 10 predictor variables, it was identification with all humanity which had the most consistent predictive power. Inasmuch as identification with all humanity has been shown to be malleable [27], future research could be designed to experimentally manipulate this belief to examine the causal effects, if any, it has on individuals’ cooperative health behavior and prosocial responding during COVID-19.

Second, research has begun on the factors that predict identification with all humanity [19], but it currently is unknown how this belief emerges ontogenetically, within an individual’s development. It has been suggested that parenting behaviors or styles may be a mechanism in the intergenerational transfer of identification with all humanity, e.g., implicit or explicit moral instruction about the equality between people who look or act different from one another [18]. Investigating how this human value is communicated to the child in different sociocultural contexts, potentially affecting the expression of prosociality [30], is an important task for future research in developmental psychology.

Third, while our data included respondents from many countries, our survey in each country was not designed to be representative of that society. Most of the current sample involved U.S. respondents, as has other work on identification with all humanity [16]. We acknowledge that our sample, like others sampled during this pandemic [31–33], is not representative in various ways (e.g., 72% of respondents had a university degree). Based on this initial study, it would be especially interesting for future work to use representative surveys in order to examine how identification with all humanity is expressed differently or predicts different constructs as a function of culture. Our analytic approach allowed us to say that the reported patterns generalize across the countries tested (see Methods and materials), but future research with larger *n*s and more representative samples would be useful for examining possible cultural variations in identification with all humanity and the behavioral sequalae in different cultures.

Fourth, our data was self-reported by respondents. Self-reports of behaviors and intentions do not necessarily correspond to actual behavior. It would be useful to measure the differential ways that individuals who score relatively high or low on identification with all humanity actually function in their daily life. It is notable that although the current results derive from respondents’ self-reports and the outcome data are skewed, e.g., cooperative health behaviors were at a mean of 89% compliance (17.76 out of a maximum score of 20, see Table 1 and Methods and materials), these patterns are similar to other self-reported data emerging from the pandemic [31–33]. Relevant to the concerns arising from the self-reported nature of the data (e.g., self-serving bias, reporting errors), it is notable that respondents’ cooperative, prosocial behavior was most consistently linked to identification with all humanity. Such specificity weighs against a self-serving bias alternative explanation, because respondents presumably had no reason to consciously manipulate their responses such that identification with all humanity (rather than identification with the nation, for example) would emerge as the strongest statistical predictor in the entire dataset. Indeed, identification with all humanity showed a very different profile than did identification with one’s own nation.

Fifth, our investigation did not measure, and hence cannot rule out, other psychological factors such as agreeableness, empathy, or universalist values. Nonetheless, the research that introduced the construct of identification with all humanity [16, 19] showed that although these other prosocial constructs were correlated with identification with all humanity, they were not redundant with it. Future work could examine the possibility that these other psychological factors (e.g., agreeableness, empathy) could work together with identification with all humanity to explain people’s intentions, choices, and behaviors during the pandemic.

We also acknowledge that many of our measures (e.g., the moral dilemmas) were used for the first time here, inasmuch as they were created in rapid response to the emerging pandemic situation. Two of the dilemmas, shopping for a family and calling an ambulance, seemed weaker than the other outcome measures (Table 2), and it may be that these moral dilemma scenarios capture prosocial behavior to a lesser extent than donating a mask or driving someone to the hospital. Future research should further seek to establish the reliability and validity of the present measures as well as that of other measures that are pre-existing and could be developed further.

The current results have implications for interdisciplinary approaches to addressing and potentially managing the COVID-19 pandemic [1, 4]. For example, it is possible, but remains to be demonstrated, that promoting peoples’ feelings of connection to all humanity through experimental interventions [27] may promote cooperative health behaviors and helpful responding during the pandemic. On average, our respondents relatively strongly identified with all humanity, at a mean score of 4.04, but there was considerable variation (*SD* = 0.83). This variation suggests that there is much room to promote identification with all humanity both among those already relatively identified and among those at the lower end of identification. From a data science and public health perspective, even identifying *who* is likely to engage cooperatively and helpfully during COVID-19 may yield important insights not only for psychological theory, as we have tried to show here, and also for policy and practice [34, 35] as global societies are confronted with humanitarian crises of both human and natural origins.

## Conclusions

In sum, our findings point to a potent psychological construct that predicts peoples’ self-alignment with global health guidelines as well as peoples’ level of prosocial, helpful behavior during a global health crisis. This finding may help scientists and health experts predict rates of compliance with cooperative health guidelines. While our study is correlational in nature, it paves the way for designed interventions to facilitate peoples’ feelings of connection, identification, and altruism toward strangers [3, 36–38]. An alteration in the perception of one’s connection to the “family of humanity” at large could result in improvements to public health and altruism during the COVID-19 pandemic as well as in future pan-human crises.

## Supporting information

S1 File GLMM analyses using countries with 10 or more participants, zero-order correlations among predictor variables, and GLMM analyses of two-way interactions with education. (PDF)
