# Supplementary material for: Identifying with all humanity predicts cooperative health behaviors and helpful responding during COVID-19
Source: PLoS One. 2021 Mar 10;16(3):e0248234. doi: 10.1371/journal.pone.0248234 (PMC7946174; doi:10.1371/journal.pone.0248234)
Supplement: S1 File — (PDF) [file pone.0248234.s001.pdf]

Further Analyses

1. Number of respondents. We also conducted the same GLMM analysis as reported in the main text (Fig 1), but with a sample restricted to those countries which had *at least* 10 respondents (N = 2343) instead of all of the countries used in the full analytic sample (N = 2537). The reduced sample included 20 countries (listed in descending order): United States, China, South Africa, Germany, United Kingdom, Philippines, India, Brazil, Spain, Canada, Taiwan, Sweden, Australia, Iran, Nigeria, Switzerland, Italy, Singapore, Chile, and Kenya. The pattern reported in the main text remains essentially unchanged (compare Fig S1 to Fig 1).

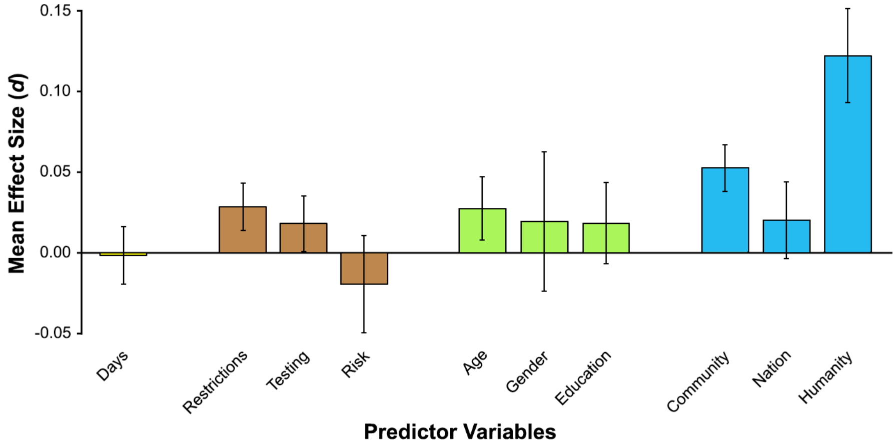

Fig S1. Mean effect sizes (estimated Cohen's *d*) across five outcomes for each predictor variable, when restricting the sample to countries with at least 10 respondents (N = 2343). The predictors are grouped into five classes (indicated by color) time in days since survey start (yellow), contextual factors (brown), respondent demographic characteristics (green), two psychological identification variables and our hypothesized predictor of identification with all humanity (blue). Positive values indicate that the predictor is associated with higher outcome scores. Error bars represent the standard error of the mean effect size.

2. Zero-order correlations among variables. Table S1 provides the correlations among the 10 variables used as predictors in the GLMM analyses.

Table S1. Zero-order correlations among variables (N = 2537).

Variables	1	2	3	4	5	6	7	8	9	10
**1. Days**	—									
**2. Restrictions**	*-0.39*	—								
**3. Testing**	*0.14*	*-0.05*	—							
**4. Risk**	*0.11*	0.01	-0.01	—						
**5. Age**	*0.15*	*0.04*	*-0.11*	*0.26*	—					
**6. Gender**	*0.05*	-0.03	-0.00	0.04	*0.04*	—				
**7. Education**	*-0.16*	*0.08*	-0.03	-0.01	*0.13*	-0.00	—			
**8. Community**	-0.00	-0.00	0.02	*0.07*	*0.13*	*0.12*	*0.06*	—		
**9. Nation**	*-0.11*	0.02	*0.06*	*0.11*	*0.09*	*0.07*	*0.05*	*0.68*	—	
**10. Humanity**	*-0.14*	*0.14*	-0.01	*0.06*	*0.04*	*0.10*	*0.07*	*0.55*	*0.59*	—

Italicized *r*-values have *P*-values less 0.05.

3. Examining two-way interactions with education. A reviewer usefully asked whether educational attainment interacted with the reported results. In the main text we report that: (i) identification with all humanity was a significant predictor of all five outcomes while simultaneously controlling for education and other variables (Fig 2) and (ii) education by itself (dichotomous: having university degree or higher vs. not) significantly predicted only one of five outcomes (Fig 2). In response to a reviewer, we added two-way interactions between education and other predictors to our GLMM model, as a Model 3, testing education interactions with age, gender, and the three psychological identification variables (identification with community, nation, all humanity). The results of Model 3 showed that identification with all humanity remained a significant predictor of each of the five outcomes ($P_{\text{range}} < 3.7 \times 10^{-22}$ to < 0.022); across all outcomes, adding two-way interactions did not substantively change the findings already reported. Of the 25 tested, only two of the education interactions were significant—an education × gender interaction for mask donation and an education × identification-with-nation interaction for mask donation. Given that 25 interactions (5 interactions with education [age, gender, community, nation, all humanity] for each of 5 outcomes) were tested, we are hesitant to further interpret this.
